# Supplementary material for: Association between oral microbiome and seven types of cancers in East Asian population: a two-sample Mendelian randomization analysis
Source: Front Mol Biosci. 2023 Nov 21;10:1327893. doi: 10.3389/fmolb.2023.1327893 (PMC10702768; doi:10.3389/fmolb.2023.1327893)
Supplement: Supplementary file 1 [file DataSheet1.ZIP › Supplementary Materials/MR plots of saliva/saliva═╝/Breast cancer/pheno.758_to_breast cancer_forest.pdf]

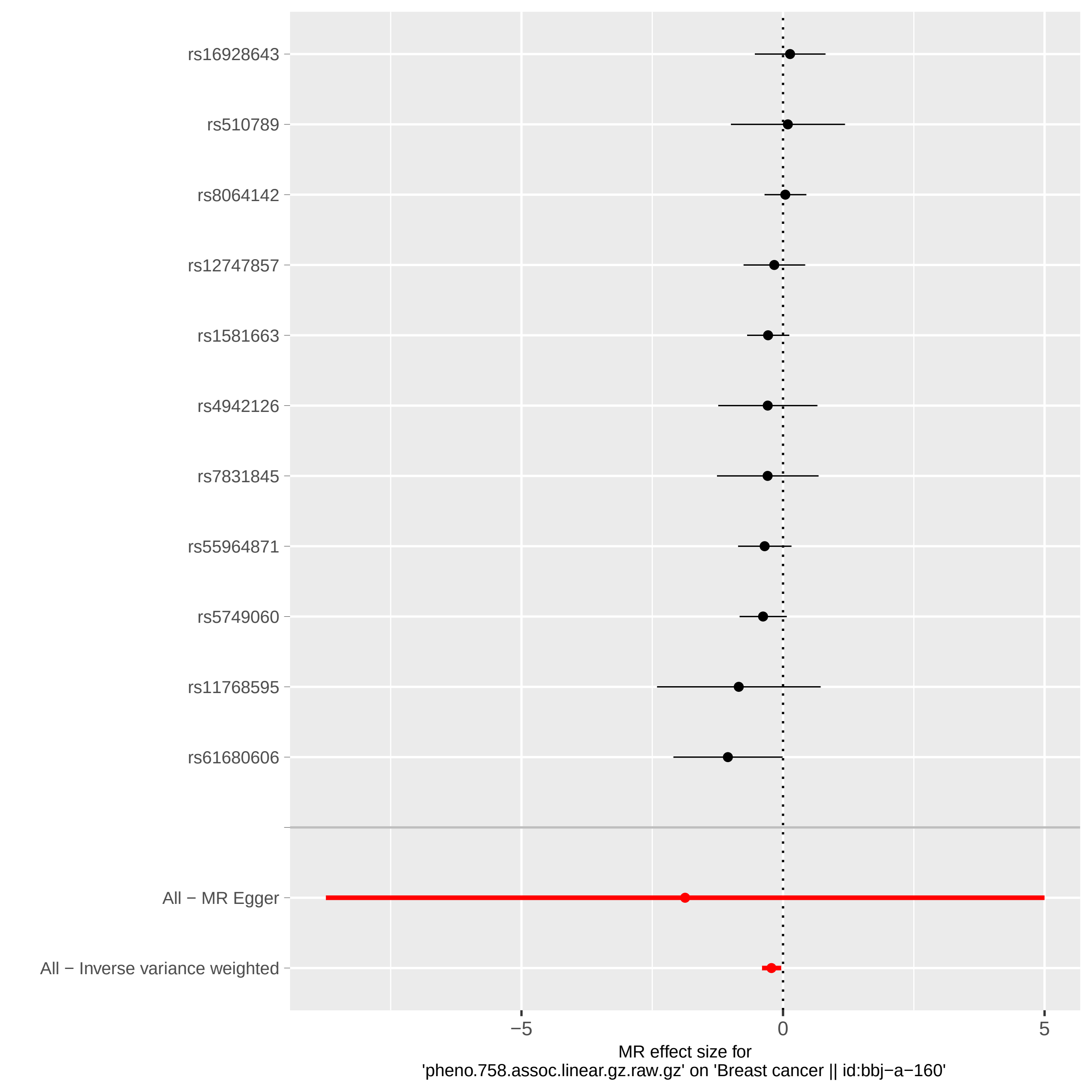
rs16928643
rs510789
rs8064142
rs12747857
rs1581663
rs4942126
rs7831845
rs55964871
rs5749060
rs11768595
rs61680606
All − MR Egger
All − Inverse variance weighted
−5
0
5
MR effect size for
'pheno.758.assoc.linear.gz.raw.gz' on 'Breast cancer || id:bbj−a−160'
